# Robust two-dimensional superconductivity and vortex system in Bi_2_Te_3_/FeTe heterostructures

**DOI:** 10.1038/srep26168

**Published:** 2016-05-17

**Authors:** Hong-Chao Liu, Hui Li, Qing Lin He, Iam Keong Sou, Swee K. Goh, Jiannong Wang

**Affiliations:** 1 Department of Physics, The Hong Kong University of Science and Technology, Clear Water Bay, Hong Kong, China; 2 Department of Physics, The Chinese University of Hong Kong, Shatin, New Territories, Hong Kong, China; 3 William Mong Institute of Nano Science and Technology, The Hong Kong University of Science and Technology, Clear Water Bay, Hong Kong, China

## Abstract

The discovery of two-dimensional superconductivity in Bi_2_Te_3_/FeTe heterostructures provides a new platform for the search of Majorana fermions in condensed matter systems. Since Majorana fermions are expected to reside at the core of the vortices, a close examination of the vortex dynamics in superconducting interface is of paramount importance. Here, we report the robustness of the interfacial superconductivity and 2D vortex dynamics in four as-grown and aged Bi_2_Te_3_/FeTe heterostructure with different Bi_2_Te_3_ epilayer thickness (3, 5, 7, 14 nm). After two years’ air exposure, superconductivity remains robust even when the thickness of Bi_2_Te_3_ epilayer is down to 3 nm. Meanwhile, a new feature at ~13 K is induced in the aged samples, and the high field studies reveal its relevance to superconductivity. The resistance of all as-grown and aged heterostructures, just below the superconducting transition temperature follows the Arrhenius relation, indicating the thermally activated flux flow behavior at the interface of Bi_2_Te_3_ and FeTe. Moreover, the activation energy exhibits a logarithmic dependence on the magnetic field, providing a compelling evidence for the 2D vortex dynamics in this novel system. The weak disorder associated with aging-induced Te vacancies is possibly responsible for these observed phenomena.

In condensed matter physics, exotic physical phenomena usually emerge at the heterostructure interface of two materials with different topological characters. Recently, new topological materials including topological insulators^1^,^2^,^3^,^4^,^5^,^6^,^7^,^8^,^9^,^10^,^11^,^12^,^13^, topological superconductors^1^,^2^,^14^,^15^,^16^,^17^, and topological semimetals^18^,^19^,^20^,^21^,^22^, have attracted considerable attention owing to the presence of novel physical properties with promising applications in spintronics, quantum computing, valleytronic devices *etc*. Unlike conventional insulators, three-dimensional (3D) topological insulators, *e.g.* Bi_2_Se_3_ and Bi_2_Te_3_, are protected by the time reversal symmetry and have insulating bulk surrounded by metallic surface states with helical Dirac fermions. At the interface between the topological surface states and an *s*-wave superconductor, a two-dimensional (2D) spinless *p*_*x*_ + *ip*_*y*_ topological superconductor was predicted to be induced by the proximity effect, which hosts the Majorana fermions^14^. In order to observe the Majorana fermions in condensed matter systems, the proximity effect has been widely investigated in topological insulator-superconductor devices^23^,^24^,^25^,^26^,^27^ and heterostructures^28^,^29^,^30^,^31^,^32^. Adopting a different strategy, we prepared and reported the 2D interfacial superconductivity in topological insulator-iron chalcogenide, *i.e.*, Bi_2_Te_3_/FeTe heterostructure, where neither Bi_2_Te_3_ nor FeTe thin films was superconducting^33^. Since superconductivity can be stabilized in a heterostructure containing the topological insulator Bi_2_Te_3_, their interplay is expected to provide a new platform for the search of Majorana fermions, which obey the non-Abelian statistics and may play an important role in the development of fault-tolerant quantum computer^34^. Furthermore, in topological superconductors the Majorana bound states are always hosted in the vortex cores^14^. Therefore, the stability of the superconductivity and vortex dynamics study become important topics for the search and further manipulation of the Majorana fermions.

Here, we study the robustness of the interfacial superconductivity and 2D vortex dynamics in the Bi_2_Te_3_/FeTe heterostructure using the electrical transport measurements. After two years’ air exposure, the aged heterostructures remain superconducting but with a broader transition region. A new feature appears at ~13 K after two years, and its relevance to superconductivity is further revealed by high field studies. The resistance of the Bi_2_Te_3_/FeTe heterostructure just below the transition temperature follows the Arrhenius relation, which we attribute to thermally activated flux flow (TAFF) behavior. The activation energy exhibits a logarithmic dependence on the applied magnetic field, indicating the existence of a 2D vortex system.

## Results and Discussion

**Figure 1:**
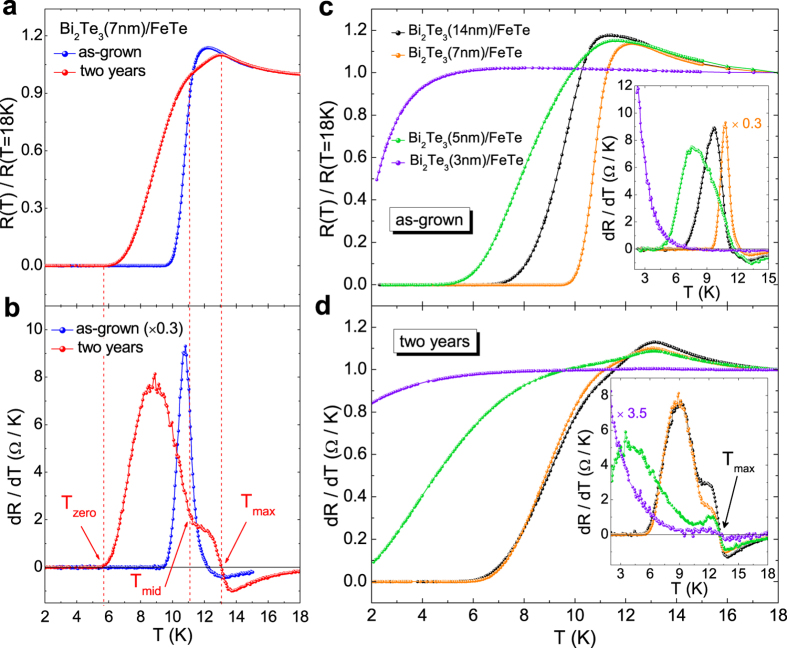
(**a**) Normalized temperature dependent resistances and (**b**) *dR*/*dT* curves of sample Bi_2_Te_3_(7 nm)/FeTe in as-grown and after-two-years cases. The as-grown and after-two-years normal state resistances at 18 K are 21.0 Ω and 25.3 Ω, respectively. The *T*_*max*_, *T*_*mid*_, *T*_*zero*_ of after-two-years case are indicated with dash lines and arrows. Normalized temperature dependent resistances of samples Bi_2_Te_3_(14 nm)/FeTe, Bi_2_Te_3_(7 nm)/FeTe, Bi_2_Te_3_(5 nm)/FeTe and Bi_2_Te_3_(3 nm)/FeTe in (**c**) as-grown and (**d**) after-two-years case, respectively. The corresponding *dR*/*dT* curves are given in their insets.

Figure 1(a) presents the normalized temperature dependent resistances *R*(*T*) of Bi_2_Te_3_(7 nm)/FeTe in both as-grown and after-two-years measurements. Before the normalization, the normal state resistance of all after-two-years samples is generally higher: using *R*(18 K) as a benchmark, it is 12–64% larger compared with the as-grown samples. Since the formation of Te vacancies is always observed in the Bi_2_Te_3_ and FeTe after long-term exposure to air^35^,^36^,^37^, it provides an explanation for the aging induced resistance increase here. Despite the increase in resistance, superconductivity is still robust after two years’ air exposure, although the transition regime becomes broader and the zero-resistance temperature *T*_*zero*_ drops. Therefore, the weak disorder from the Te vacancies is not completely detrimental to superconductivity; instead it provides an interesting avenue for investigating the vortex dynamics, which will be discussed later. Compared with the as-grown result, in Bi_2_Te_3_(7 nm)/FeTe the temperature of the maximum resistance, *T*_*max*_, shifts from 12.4 K to 13 K and the resistance exhibits a two-step drop below *T*_*max*_ in the after-two-years case. To provide a clearer view on these results, *dR*/*dT* curves are plotted in Fig. 1(b). As can be seen, in the after-two-years case, a shoulder appears at around 11–13 K, corresponding to the first slow resistance drop in the *R*(*T*) curve. As temperature further decreases, a sharp transition starts. We define the starting point of the sharp resistance drop as *T*_*mid*_ in after-two-years *R*(*T*) result, as shown in Fig. 1(b). Therefore, compared with the as-grown result, it demonstrates that an additional new feature appears at around 13 K in after-two-years case.

To learn more about the observation of the new feature, we further measure *R*(*T*) of after-two-years heterostructures with different Bi_2_Te_3_ layer thicknesses. Compared with the as-grown results in Fig. 1(c) and its inset, a broader superconducting transition and a two-step resistance drop are observed in all after-two-years heterostructures even with the Bi_2_Te_3_ layer down to 3 nm as shown in Fig. 1(d) and its inset. Furthermore, from the *dR*/*dT* curves in the inset of Fig. 1(d), *T*_*max*_ shows an increase after two years and locates at ~13 K for all samples, manifesting that the new feature is indeed induced by the aging effect. Meanwhile, relative to the full superconducting transition, the shoulder is weakened as the Bi_2_Te_3_ thickness decreases as shown in the inset of Fig. 1(c,d), which indicates that the new feature around 13 K is probably relevant to the interfacial superconductivity of the heterostructure.

**Figure 2:**
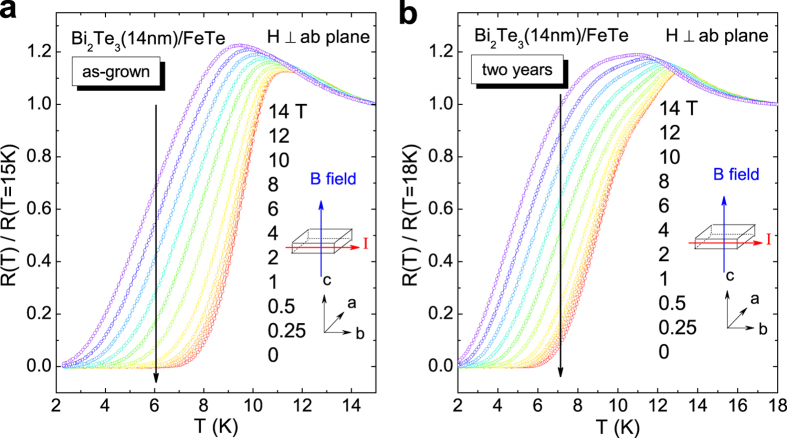
Normalized temperature dependent resistances of sample Bi_2_Te_3_(14 nm)/FeTe in magnetic fields ranging from 0 T to 14 T in (**a**) as-grown and (**b**) after-two-years cases, respectively. The magnetic field is perpendicular to the *ab* plane as shown in the insets.

**Figure 3:**
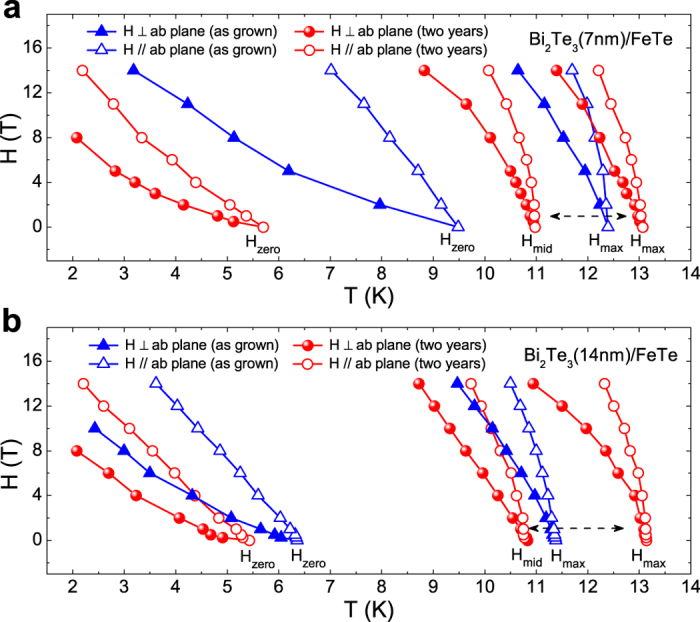
Magnetic field-temperature phase diagram of samples (**a**) Bi_2_Te_3_(7 nm)/FeTe and (**b**) Bi_2_Te_3_(14 nm)/FeTe. The upper critical field *H*_*max*_ and *H*_*zero*_ in the as-grown case are plotted as triangle symbols, and the *H*_*max*_, *H*_*mid*_ and *H*_*zero*_ in the after-two-years case are presented as circle symbols. The solid and hollow symbols represent the perpendicular and parallel fields’ situations, respectively.

To further investigate the aging effect on the superconducting transition of Bi_2_Te_3_/FeTe heterostructure, the temperature dependent resistances in different magnetic fields are studied. Fig. 2(a,b) show the as-grown and after-two-years *R*(*T*) results of Bi_2_Te_3_(14 nm)/FeTe in different magnetic fields applied perpendicular to the *ab* plane, respectively. With an increasing magnetic field, *T*_*max*_, *T*_*mid*_ and *T*_*zero*_ gradually shift to lower temperature together as superconductivity is suppressed. Similar behavior is also observed in the parallel fields. From the *R*(*T*) curves in different magnetic fields, the corresponding upper critical fields *H*_*max*_, *H*_*mid*_ and *H*_*zero*_ in both as-grown and after-two-years cases can be obtained, and the *H* - *T* phase diagrams of Bi_2_Te_3_(7 nm)/FeTe and Bi_2_Te_3_(14 nm)/FeTe are plotted in Fig. 3(a,b), respectively. For both samples, the phase diagram clearly exhibits the anisotropy between the parallel and perpendicular field for both as-grown and after-two-years cases. Meanwhile, the anisotropy ratio 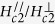 of the after-two-years sample shows a decrease, especially for *H*_*zero*_, comparing with the as-grown one. For both field directions, the as-grown *H*_*max*_ locates between the after-two-years *H*_*max*_ and *H*_*mid*_, and all three curves show the same variation trend as magnetic field changes. It further manifests that the new feature at 13 K, *i.e.*, the after-two-years *H*_*max*_, are relevant to the interfacial superconductivity of the heterostructure. In the study of BiS_2_-based superconductor LaO_0.5_F_0.5_BiS_2_, two-step drop of *R*(*T*) in different magnetic fields was also observed^38^. Since the LaO_0.5_F_0.5_BiS_2_ sample was polycrystalline, the origin of two anisotropic upper critical fields was attributed to the anisotropy of the grains in different directions^38^. However, this scenario cannot be applied easily to our heterostructures, since they are all composed of single crystallined films. In addition, the anisotropic behavior of upper critical fields in our heterostructures can be largely suppressed and affected by the annealing process in N_2_ atmosphere, at the expense of lowering the superconductivity transition temperature (see Supplementary information).

**Figure 4:**
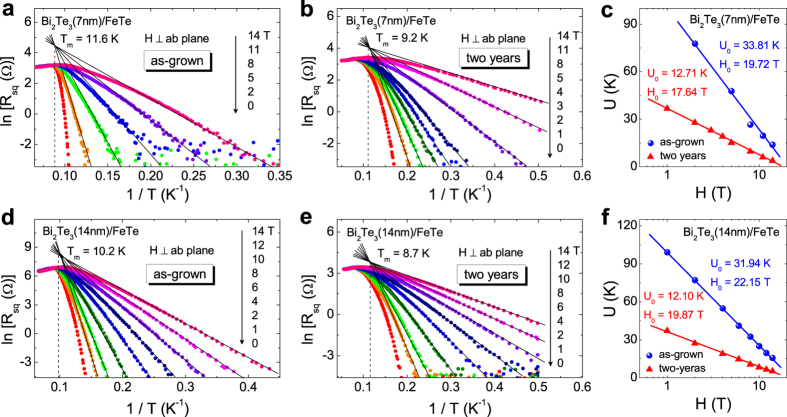
ln*R*_*sq*_(*T*) vs. 1/*T* in different perpendicular magnetic fields for sample (**a**) Bi_2_Te_3_(7 nm)/FeTe in as-grown case, (**b**) Bi_2_Te_3_(7 nm)/FeTe in after-two-years case, (**d**) Bi_2_Te_3_(14 nm)/FeTe in as-grown case, (**e**) Bi_2_Te_3_(14 nm)/FeTe in after-two-years case. The solid lines in (**a,b,d,e**) are fitting results from the Arrhenius relation, whose slopes give the values of *U* in (**c**,**f**). The solid lines in (**c**,**f**) are fitting results from the function *U* = *U*_*0*_ ln(*H*_0_/*H*).

Comparing with type-I superconductors, our Bi_2_Te_3_/FeTe heterostructure samples show a relatively broad superconducting transition regime (>3 K) in magnetic fields even for the as-grown samples. This means the mixed states exist in the transition regime and vortex dynamics studies will be important and can provide useful information about the interfacial superconducting behavior. Thermally activated flux flow (TAFF) describes the motion of vortices due to the activation over some energy barriers, *e.g.* pinning centers^39^,^40^,^41^. It was widely studied in 3D iron-based superconductors, such as *β*-FeSe single crystal^41^, Fe_1.03_Te_0.55_Se_0.45_^42^, Fe_1.14_Te_0.91_S_0.09_^43^, NdFeAsO_0.7_F_0.3_^44^ and cuprates superconductors, such as, Bi_2_Sr_2_CaCu_2_O_8+δ_^45^ and YBa_2_Cu_3_O_7-δ_^46^. Recently, TAFF was also reported in novel 2D superconductors, such as FeSe single layer^47^ and exfoliated NbSe_2_^48^, where in the latter case the TAFF behavior was reported to come from the unbinding of vortex-antivortex pairs. According to the TAFF theory, the resistivity *ρ* in the TAFF region follows the Arrhenius relation^39^,^40^,^41^


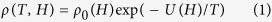


where *ρ*_*0*_ is a temperature independent constant, *U* is the thermal activation energy of the flux flow. Therefore, Eq. (1) can be written as ln *ρ*(*T, H*) = ln *ρ*_*0*_(*H*) − *U*(*H*)/*T*. At a fixed magnetic field, ln *ρ*(*T*) − 1/*T* plot is expected to have a linear relation in the TAFF regime. Two samples, Bi_2_Te_3_(7 nm)/FeTe and Bi_2_Te_3_(14 nm)/FeTe, are carefully studied by applying the TAFF theory, as shown in Fig. 4. For both as-grown and after-two-years cases, the temperature dependent resistances of two samples in different perpendicular fields are plotted on ln *ρ*(*T*) − 1/*T* axes in Fig. 4(a,b,d,e), respectively. As can be seen, all curves exhibit good linear behaviors at low temperature region, manifesting that they follow the Arrhenius relation very well. All fitting lines in different fields cross to one point, whose corresponding temperature *T*_*m*_ should be equal to the *T*_*c*_ of the system. For the as-grown case, *T*_*m*_ of Bi_2_Te3(7 nm)/FeTe and Bi_2_Te_3_(14 nm)/FeTe are obtained as 11.6 K and 10.2 K, which are close to their *T*_*max*_ = 12.4 K and 11.4 K, respectively. However, for after-two-years results (*c.f.* Fig. 4(b,e)), *T*_*m*_ of Bi_2_Te_3_(7 nm)/FeTe and Bi_2_Te_3_(14 nm)/FeTe are obtained as 9 K and 8.7 K, which are closer to their *T*_*mid*_ of ~ 11 K than *T*_*max*_ of ~ 13 K. This indicates that as-grown samples fall into the TAFF region much faster than the after-two-years samples when the superconducting transition commences. This slower approach to the TAFF region in the after-two-years samples, to a large extent, is affected by the new feature around 13 K, although the origin of this new feature remains unclear.

From the linear fitting of ln *ρ*(*T, H*) − 1/*T* curves, the activation energy *U*(*H*) can be obtained from the slope value. Figure 4(c,f) displays *U* at different magnetic fields for Bi_2_Te_3_(7 nm)/FeTe and Bi_2_Te_3_(14 nm)/FeTe, respectively. For the as-grown samples (triangular symbols in Fig. 4(c,f)), *U* exhibits a logarithmic dependence on the magnetic field, *U* = *U*_*0*_ ln(*H*_0_/*H*), with *H*_*0*_ ≈ *H*_c2_, as observed in other 2D systems^48^,^49^,^50^. This observation of a 2D vortex system is consistent with the earlier report of the Berezinsky-Kosterlitz-Thouless transition in the Bi_2_Te_3_/FeTe interface^33^,^51^. For both Bi_2_Te_3_(7 nm)/FeTe and Bi_2_Te_3_(14 nm)/FeTe samples, the fitted value of *H*_0_ decreases after two years’ air exposure as displayed in Fig. 4(c,f), which shows a good agreement with the result of upper critical field *H*_*zero*_ in Fig. 3. The energy prefactor 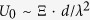, where *d* is the superconducting layer thickness, *λ* is the penetration depth and 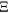 is a numerical factor which depends on the nature of the energy barrier^50^. In the aged samples, *U* shows an overall decrease, implying that the aging weakens the vortex pinning behavior or enhances the flux flow of the system. Interestingly, the logarithmic dependence of *U* on *H* remains valid, albeit with a lower *U*_0_ (circular symbols in Fig. 4(c,f)). Therefore, the 2D nature of the vortex system remains robust in the aged heterostructures. Assuming that the dominant thermal activation mechanism remains the same in the aged samples, the drop in *U*_0_ compared with the as-grown samples can be attributed to an increase in *λ*. Empirically, *λ*(0) ≈ 1.05×10^−3^(*ρ*_0_/*T*_*c*_)^1/2^, where *ρ*_0_ is the residual resistivity of the normal state and *λ*(0) is the zero temperature limit of the penetration depth^52^. Further assuming that the temperature dependences of *λ*(*T*) and *ρ*(*T*) do not vary strongly with age, we can estimate 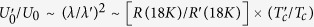, where the primed quantities are for the aged sample. For Bi_2_Te_3_(7 nm)/FeTe, take 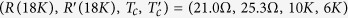, 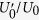 is estimated to be ~0.50, in reasonable agreement with the observed ratio of ~0.38. Following the same procedure for Bi_2_Te_3_(14 nm)/FeTe, 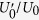 is estimated to be ~0.52 whereas the observed ratio is also ~0.38.

One possible scenario responsible for the logarithmic magnetic field dependence of *U* is the nucleation and the subsequent motion of the dislocation pairs associated with the vortex lattice. In this model, *U* is primarily the energy cost of nucleating the pair^49^,^50^. In the aged samples, the lowering of *U* can be associated with the relative ease of nucleating the dislocation pairs. Since exposing Bi_2_Te_3_ and FeTe to air inevitably promotes the formation of Te vacancies^35^,^36^,^37^, the excess vacancies thus lower the energy barrier required to nucleate dislocation pairs. In addition, these vacancies introduce weak disorder to the material system, thereby resulting in a lower superconducting transition temperature and a higher normal state resistance; these trends are fully consistent with experimental observation in all aged Bi_2_Te_3_/FeTe heterostructures.

## Conclusion

In conclusion, we study the superconducting properties of as-grown and aged Bi_2_Te_3_/FeTe heterostructures. Superconductivity is robust after two years’ air exposure, even when the thickness of the Bi_2_Te_3_ layer is down to 3 nm. Comparing with the upper critical fields of the as-grown measurements, a new feature around 13 K induced by the aging effect is demonstrated to be relevant to the interfacial superconductivity. The resistance of the Bi_2_Te_3_/FeTe heterostructures below the superconducting transition temperature obeys the Arrhenius relation, which demonstrates the TAFF behaviour. The activation energy *U*(*H*) follows a logarithmic dependence on the applied magnetic field in the as-grown samples, indicating that the vortex system is two-dimensional. The logarithmic dependence remains valid in the aged samples, although *U*(*H*) becomes lower at all magnetic fields studied, leading to the conclusion that the 2D vortex system in Bi_2_Te_3_/FeTe heterostructures is robust.

## Methods

The Bi_2_Te_3_/FeTe heterostructure samples used in the experiment were grown by molecular beam epitaxy on a (111) semi-insulating GaAs substrate with an undoped 50 nm-thick ZnSe buffer layer. A 140 nm-thick FeTe layer was first deposited onto the ZnSe buffer, followed by a growth of the Bi_2_Te_3_ layer on the FeTe layer via van der Waals expitaxy. The thicknesses of the Bi_2_Te_3_ epilayer were 3 nm, 5 nm, 7 nm and 14 nm for four different wafers, respectively. Detailed structural characterizations can be found in the early work^33^. Silver paste and aluminum wires were employed to serve as the electrical contacts, after the wafers were cut into 2 mm × 6 mm strips by a diamond scribe. After the first round measurements on the as-grown heterostructures, all samples were exposed to the air atmosphere at room temperature for two years. To avoid the complication from the sample dependence, all four samples, *i.e.* Bi_2_Te_3_(3 nm)/FeTe, Bi_2_Te_3_(5 nm)/FeTe, Bi_2_Te_3_(7 nm)/FeTe, and Bi_2_Te_3_(14 nm)/FeTe, which were measured in the second round after two years, are exactly the same strips as the ones used in the first round. All transport measurements were conducted in a Quantum Design physical property measurement system with a 14-Tesla superconducting magnet and a base temperature of 2 K.

## Additional Information

**How to cite this article**: Liu, H.-C. *et al*. Robust two-dimensional superconductivity and vortex system in Bi_2_Te_3_/FeTe heterostructures. *Sci. Rep.* **6**, 26168; doi: 10.1038/srep26168 (2016).

## Supplementary Material

Supplementary Information
