# Supplementary material for: Robust two-dimensional superconductivity and vortex system in Bi2Te3/FeTe heterostructures
Source: Sci Rep. 2016 May 17;6:26168. doi: 10.1038/srep26168 (PMC4868974; doi:10.1038/srep26168)
Supplement: Supplementary Information [file srep26168-s1.pdf]

Supplementary information for “Robust two-dimensional superconductivity and vortex system in Bi_2Te_3/FeTe heterostructures”

Hong-Chao Liu[1,2], Hui Li[1], Qing Lin He[3], Iam Keong Sou[1,3], Swee K. Goh[2,*],

and Jiannong Wang[1,3,*]

[1]*Department of Physics, The Hong Kong University of Science and Technology,*

Clear Water Bay, Hong Kong, China

[2]*Department of Physics, The Chinese University of Hong Kong,*

Shatin, New Territories, Hong Kong, China

[3]*William Mong Institute of Nano Science and Technology,*

The Hong Kong University of Science and Technology, Clear Water Bay, Hong Kong, China

**corresponding author: skgoh@phy.cuhk.edu.hk, phjwang@ust.hk*

The annealing effect on Bi_2Te_3(7 nm)/FeTe samples are studied. Here, Bi_2Te_3(7 nm)/FeTe # S1 and #S2 samples come from the same wafer as the one in the manuscript after two years' air exposure but without as-grown measurements. Figure S1(a) shows the $R(T)$ curves of sample #S1 annealing in the N_2 atmosphere at 100 ^oC with different time. The corresponding dR/dT curves are plotted in Figure S1(b). From Figure S1(b), we can see that a shoulder-like structure appears around 11 K – 13.5 K in dR/dT curve without the annealing process, which is consistent with the result in the manuscript. By increasing the anneal time and holding the temperature at 100 ^oC, this shoulder-like structure weakens gradually. Furthermore, the shoulder-like structure is completely suppressed after 160 mins annealing holding at 100 ^oC. Meanwhile, as the annealing time increases, the transition temperature continues to decrease and a new T_{mid} with an unknown origin appears around 9 K, as shown in Figure S1(a) and (b). Moreover, the heterostructure seems to reach a stable state after 160 mins anneal at 100 ^oC, because a further time increase from 160 to 1060 mins gives nearly overlapped $R(T)$ curves.

Although the heterostructure can still reach a completely superconducting state above 4 K after 10 hours annealing at 100 ^{o}C, a higher annealing temperature will strongly suppressed its superconductivity. As shown in Figure S1(c), after the anneal at 160 ^{o}C around 2.5 hours, the transition temperature of sample #S2 decreases below 7 K and cannot fully develop into the superconducting state above 2 K. Our annealing effect study suggests that the micro/nano device fabrication, for example, the photolithography or electron beam lithography, which cannot avoid the high temperature processes, may do harm to the superconductivity of the Bi_2Te_3/FeTe heterostructure. This also gives an explanation that why we used diamond scribe to prepare the samples and used silver paste to achieve the wire connections in our experiments.

The annealing effect on the flux flow behavior is also studied on Bi_2Te_3(7 nm)/FeTe #S1, as shown in Fig. S2. For both before and after annealing situations, the temperature dependent resistance follows the Arrhenius relation (*c.f.* Fig. S2 (a) and (b)), and the activation energy $U(H)$ follows the logarithmic dependence on the applied magnetic field (*c.f.* Fig. S2 (c)). This indicates that the 2D vortex system is still robust after the annealing process although the value of U_0 decreases and the flux flow behavior of the system is enhanced.

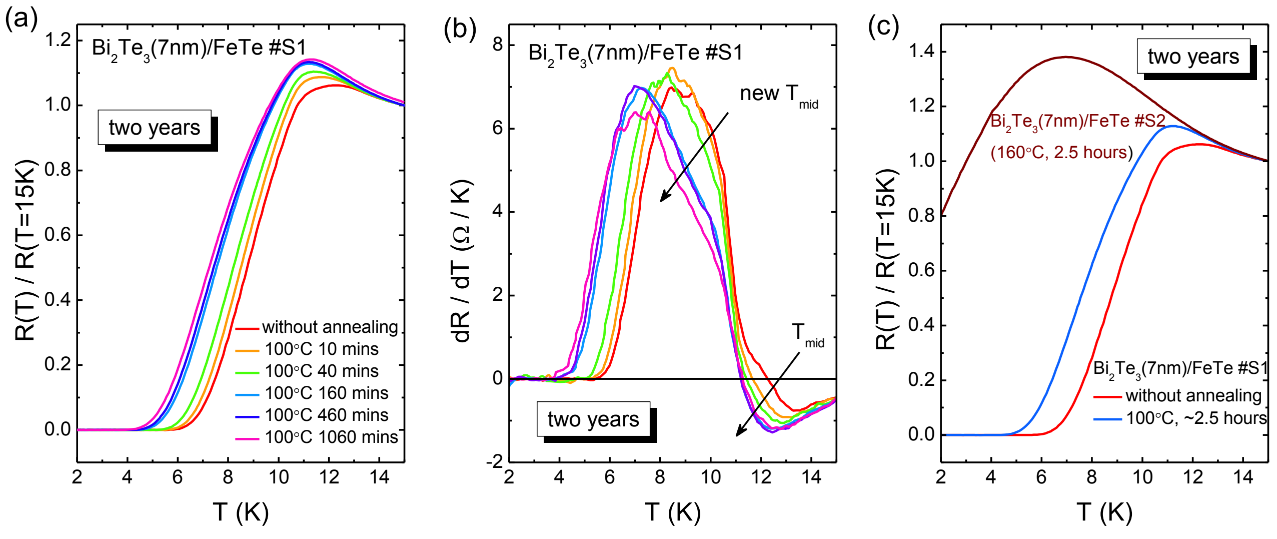

Figure S1. Annealing effect on after-two-years sample Bi_2Te_3(7 nm)/FeTe #S1 and #S2. (a) Sample Bi_2Te_3(7 nm)/FeTe #S1 annealed in the N_2 atmosphere at 100 ^oC with different time. (b) dR/dT curves corresponding to the $R(T)$ curves in (a). (c) Sample Bi_2Te_3(7 nm)/FeTe #S1 and #S2 annealed in the N_2 atmosphere at 100 ^oC 2.5 hours, and 160 ^oC 2.5 hours, respectively.

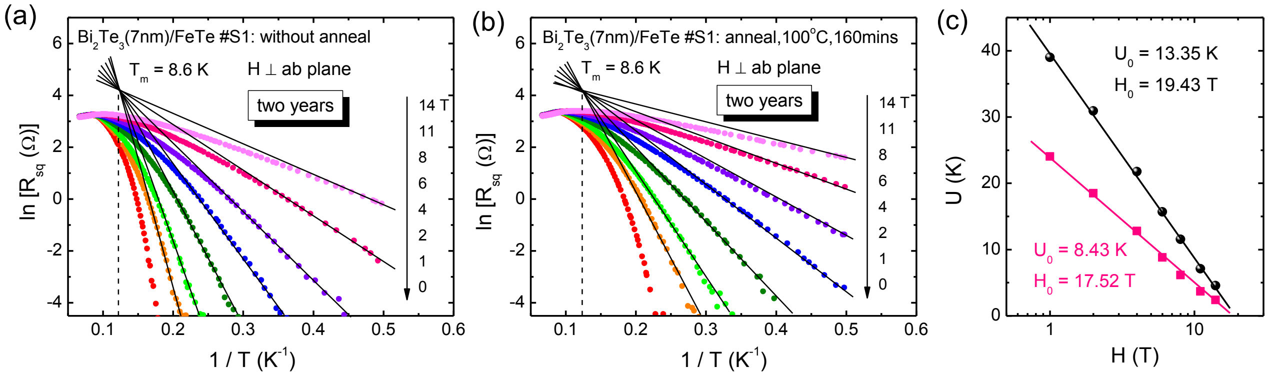

Figure S2. $\ln R_{sq}(T)$ vs. $1/T$ in different perpendicular magnetic fields for sample Bi_2Te_3(7 nm)/FeTe #S1 (a) without anneal; (b) anneal at 100^oC, 160 mins. The solid lines in (a), (b) are fitting results from the Arrhenius relation, whose slopes give the values of U in (c). The solid lines in (c) are fitting results from the function $U = U_0 \ln(H_0 / H)$.
